# From analytic to synthetic-organizational pluralisms: A pluralistic enactive psychiatry

**DOI:** 10.3389/fpsyt.2022.981787

**Published:** 2022-09-27

**Authors:** Christophe Gauld, Kristopher Nielsen, Manon Job, Hugo Bottemanne, Guillaume Dumas

**Affiliations:** ^1^Department of Child Psychiatry, Hospices Civils de Lyon, Grenoble, France; ^2^Institut des Sciences Cognitives Marc Jeannerod, UMR 5229 CNRS and Université Claude Bernard Lyon 1, Paris, France; ^3^School of Psychology, Te Herenga Waka - Victoria University of Wellington, Wellington, New Zealand; ^4^Institut Jean Nicod, École Normale Supérieure-EHESS, Paris, France; ^5^Paris Brain Institute - Institut du Cerveau (ICM), Institut National de la Santé et de la Recherche Médicale (INSERM), Center for the National Scientific Research (CNRS), APHP, Pitié-Salpêtrière Hospital, DMU Neuroscience, Sorbonne University, Paris, France; ^6^Department of Psychiatry, Pitié-Salpêtrière Hospital, DMU Neuroscience, Sorbonne University, Assistance Publique-Hôpitaux de Paris (AP-HP), Paris, France; ^7^Department of Philosophy, Sorbonne University, SND Research Unit, Center for the National Scientific Research (CNRS), UMR 8011, Paris, France; ^8^CHU Sainte-Justine Research Center, Department of Psychiatry, Université de Montréal, Montréal, QC, Canada; ^9^Mila – Québec Artificial Intelligence Institute, Université de Montréal, Montréal, QC, Canada

**Keywords:** psychiatry, enaction, cognitive science, enaction and embodied cognition, pluralism, neuroscience

## Abstract

**Introduction:**

Reliance on sole reductionism, whether explanatory, methodological or ontological, is difficult to support in clinical psychiatry. Rather, psychiatry is challenged by a plurality of approaches. There exist multiple legitimate ways of understanding human functionality and disorder, i.e., different systems of representation, different tools, different methodologies and objectives. Pluralistic frameworks have been presented through which the multiplicity of approaches in psychiatry can be understood. In parallel of these frameworks, an enactive approach for psychiatry has been proposed. In this paper, we consider the relationships between the different kinds of pluralistic frameworks and this enactive approach for psychiatry.

**Methods:**

We compare the enactive approach in psychiatry with wider analytical forms of pluralism.

**Results:**

On one side, the enactive framework anchored both in cognitive sciences, theory of dynamic systems, systems biology, and phenomenology, has recently been proposed as an answer to the challenge of an integrative psychiatry. On the other side, two forms of explanatory pluralisms can be described: a non-integrative pluralism and an integrative pluralism. The first is tolerant, it examines the coexistence of different potentially incompatible or untranslatable systems in the scientific or clinical landscape. The second is integrative and proposes to bring together the different levels of understanding and systems of representations. We propose that enactivism is inherently a form of integrative pluralism, but it is at the same time a component of the general framework of explanatory pluralism, composed of a set of so-called analytical approaches.

**Conclusions:**

A significant number of mental health professionals are already accepting the variety of clinical and scientific approaches. In this way, a rigorous understanding of the theoretical positioning of psychiatric actors seems necessary to promote quality clinical practice. The study of entanglements between an analytical pluralism and a synthetic-organizational enactivist pluralism could prove fruitful.

## Introduction

The field of clinical and scientific psychiatry deals with a vast spectrum of phenomena, from subjective experiences and social dynamics to brain activity and computational models, or psychotherapies and pharmacological treatments. By nature then, psychiatry is a discipline requiring a plurality of explanations and perspectives. Indeed, a patient seen in consultation can be understood in genetic, neurological, cognitive, psychoanalytical, developmental, and/or socio-cultural terms (1). As a result, reliance on sole *reductionism*, whether explanatory, methodological or ontological, is difficult to support in clinical psychiatry. Faced with the multiplicity of perspectives and kinds of explanations observed in clinical practice, conceiving that a single explanation or perspective can summarize the patient and her/his subjectivity is a view largely abandoned in the literature and in the field (2, 3). Anti-reductionist approaches have played an interesting role in deconstructing this conception (4). The notion of emergence in particular allows for a move away from a reductionist perspective. Consider for example the notion of supervenience, developed especially by Jaegwon Kim, which conceives that phenomena at higher scales depend those beneath them, without being reducible to it (5), e.g., a beautiful painting depends on the physical qualities of the paint in that it reflects certain wave-lengths of light, but this dependence says almost nothing about why the painting is beautiful. Such strongly *anti-*reductionistic approaches however, provide few practical answers to understand and support heterogeneous and multi-perspectivist daily clinical practice.

In contrast, the acceptance of all explanations and perspectives without weighing the value of each seems irrelevant. Such a *radical holism* would consider each perspective with the same value as another (e.g., the psychoanalytic explanation explains acoustico-verbal hallucinations as well as the neurobiological explanation) and would require that all these perspectives be systematically considered (e.g., the psychoanalytical approach and the neurobiological approach must be necessarily considered for any psychiatric phenomenon). For these reasons, this kind of holism seems also untenable in clinical psychiatry.

It is in this context that explanatory pluralism has come to be seen as relevant for psychiatry. Explanatory pluralism constitutes a general epistemological framework, an *umbrella term*, under which it is accepted that there are multiple ways to explain the world, and in the case of psychiatry, multiple ways to explain our patients presenting life problems and our ways of treating them. In other words, what is plural under explanatory pluralism are the explanations themselves – we are left free to utilize multiple simultaneous explanations of any type (e.g., mechanistic, causal, dynamic, etc.), drawing from any perspective, system of representation, or scientific/clinical method, so long as it adds sufficient explanatory or pragmatic value. Explanatory pluralism thus allows us to hybridize various clinical practices – e.g., pharmacological treatment and psychotherapy – despite the fact that such practices are often grounded in different kinds of explanations of what is happening to the patient (e.g., neurochemical vs. cognitive-behavioral). As a general epistemological framework, explanatory pluralism allows multiple explanations to co-exist, facilitating a flexible use of various evidence-based/well-reasoned clinical practices even when the underlying explanations may not completely align (while also considering other constraints of practice such as client preferences, ethics, policy, etc.). Such an explanatory pluralism, in which several kinds of explanations are mobilized, e.g., neuroscientific and psychoanalytical, goes hand in hand with: (i) an ontological pluralism, positioned on the metaphysical level and for which there are several objects in the world according to the explanations, e.g., brains and *Dasein* both exist, (ii) and methodological pluralism, in which a variety of tools and treatments are used, e.g., genetic testing and interviewing, psychotherapy, and pharmacotherapy. Explanatory pluralism at the explanatory level can thus be seen as entailing an ontological pluralism and conditions a pluralism at the level of methods (both clinically and in research).

Psychiatry can thus be understood as engaging with explanatory pluralism. Many attempts claiming (more or less explicitly) such explanatory pluralism have been conducted in the history of psychiatry, such as the so-called clinical “integrative” approaches (6). Scientific disciplines as diverse as molecular genetics, biochemistry and neurobiology have been integrated into this explanatory pluralism, e.g., through the Research Domain Criteria (RDoC) project (7). More recently, computational sciences have grafted themselves onto this dynamic of explanatory pluralism in psychiatry (8). They propose to model a variety of levels of understanding of living organisms (7) within statistical models (such as symptom network models) (9, 10), for instance with Bayesian models (11).

Recognition of this pluralism in psychiatry is sometimes related to the Engel's biopsychosocial model (12), although this relationship was maybe not intended initially (13). The biopsychosocial model was described by Pascal Engel in 1977 as a representation of the human being in which biological, psychological, and social factors are considered to participate simultaneously in the maintenance of health or the development of disease (12). Although adopted by a large number of clinicians, this model has been the target of numerous criticisms since its development, criticisms coming jointly from the philosophy of science and clinical psychiatry. The biopsychosocial model, at least in its initial version (14), constitutes a juxtaposition of three levels of analysis (biological, psychological, and social), randomly chosen and vaguely described according to a systems theory transposed from physics (13, 15). Moreover, in clinical practice and in research, this model is disappointing because it does not give equal weight to these three levels – the American psychiatrist Steven Sharfstein, in his inaugural speech of his presidency of the APA, thus argued that it was in practice a “bio-bio-bio” approach (16). The biopsychosocial model in its initial form has artificially clear boundaries, without any real attempts at integration, causality, or communication between these levels (17, 18). In particular, due to the absence of mutual causality between the levels (19), this model does not consider the first-person experience of the psychiatric phenomena, nor the meaning that individuals give to their existence (or to others and to the world).

A more recent approach for considering the integrated or interwoven nature of causes in psychiatry is the enactive approach (13, 20). Enactivism, not reducible to psychiatry, is a philosophy of mind approach of human functioning rooted in systems biology, dynamical systems theory, cognitive sciences, and phenomenology (21). This approach is based on the idea that cognition is an embodied activity that is *enacted* through the interaction of an organism with its environment. Instead of the generally received hierarchical and brain-centric view whereby chemical structures are organized into neurons and neurons are organized into neural circuits, and these structures, in turn, are seen to ‘process' the world *via* sensory input, enactivism represents a much more *loopy view* (22). Indeed, under enactivism it is understood that biological objects such as neural circuits do not simply “cause” behaviors, rather they are one part of a wider network of causal chains that simultaneously cause and are caused by behavior. These causal chains are themselves constrained and influenced by other parts of the organism as well as its wider dynamics and intentions. Enactivism focuses on the biologically constituted organism standing in relation to the world. Thus, to understand behavior, we need to consider the wider brain-body-environment system evolving over time (22–26). Another important facet of enactivism refers to the central role given to meaning and experience. Regarding the first, cognition is the act of making sense of the world (including oneself), often referred to as *sense-making*. Regarding the second, phenomenological experience is not understood as a product of the brain, but as the world from the concerned point of view of a self-maintaining and adaptive organism (26).

Given the complexity that the enactive approach demands to be reckoned with, it has been argued that enactivism entails a kind of pluralism – that under an enactive understanding of psychiatry there should be many different legitimate ways to explain mental disorders (24, 27, 28). However, the kind of explanatory pluralism entailed by enactivism is different to the general explanatory pluralism discussed above, as we will develop. In this article, we will show that the general pluralist framework is a much broader epistemological construction than enactivism. We will argue that they are of different statuses since the first is a general epistemological framework while the second is an approach to conceptualizing human functioning.

Therefore, as a theoretical approach that does not *itself* provide tools for exploring all relevant mechanisms (e.g., neuropsychological), enactivism would be more restricted than explanatory pluralism for the clinical practice of psychiatry. Enactivism would be only one of the approaches contained within a general framework of pluralism, albeit a very useful and integrative one.

In this article, we compare the general framework of explanatory pluralism and the enactivist approach. Although psychiatry can be understood as both a clinical activity and a research activity, in this article, we are focused on psychiatry as a clinical activity. Indeed, we seek to identify a perspective in which the clinician in psychiatry places himself, and more generally any individual who is interested in psychiatry. We question the methodological and pragmatic gain that each of these approaches brings to clinical psychiatry. First, we show the advantages of the enactive approach for clinical psychiatry, by analyzing how it can be conceptually and methodologically used in pedagogy and clinical practice. Secondly, we consider the different kinds of explanatory pluralism applied to psychiatry, detailing its clinical, pedagogical, and theoretical implications. Finally, in the third part, we discuss the issues of the relations between the general and philosophical framework of explanatory pluralism and the enactive approach, in clinical psychiatry. This third part aims to explore the challenges and benefits of crossing an explanatory pluralist framework and the enactive approach. The paper is neutral on the point of whether enactivism should be seen as part of a pluralistic approach or whether pluralistic methods can be understood beneath a wider enactive frame.

## The enactive stance

### The enactive approach, an embodied cognition

Enactivism is based jointly on phenomenology (a philosophical discipline centered on the analysis of the experience lived by a subject), theory of dynamic systems (a mathematical discipline studying the laws applied to the evolution of a system), and systems biology (a biological discipline seeking to integrate different levels of biological information to understand the functioning of an organism). It seeks to provide an approach for understanding human behavior and subjective phenomena, such as belief or perception, based on a set of principles which we will review in the following section.

The enactivist approach is initially based on the idea of *autopoiesis* (21, 29, 30), an observation within cellular biology that cells represent self-maintaining and adaptive, operationally-closed systems (31), capable of coupling and changing through the interaction with their environment. In *The Embodied Mind* published in 1991, Francisco Varela, Evan Thompson, and Eleonor Rosch (21) hypothesized that this concept of autopoiesis was a fitting analogy for the mind and could be used to ground a new approach to philosophy of mind and the mind sciences.

These authors sought to move away from an understanding of the mind grounded in a metaphor of computation and representation, and instead understand the “mind” through analogy to life forms, especially notions of biological autonomy and coupling. Under this approach, they proposed cognition is a relational process that is *enacted* through the *embodied* interaction of an organism *embedded* in the world. This formulation comes in response to the questions provoked by the growing explanatory gap between cognitive sciences and phenomenology, the former often finding themselves unable to transcribe, explain, or represent the subjective reality experienced in the first person by an individual. A branch of phenomenology known as neurophenomenology is related to but differs from the enactive approach and seeks above all to address the hard problem of consciousness at the crossroads of neuropsychology, neuro-anthropology and behavioral neuroscience. The enactive approach recognizes that cognitive activities are carried out by organisms in constant interaction with their environment. This assumption ensures that individuals and environments continually co-construct each other, the action of the former drastically influencing the nature of the latter, and vice versa. This formulation contrasts with the dated traditional cognitivist view according to which the brain forms a fixed and immutable representative cartography, i.e., an internal model which would replicate the world, as a mirror of sensory reality (32). For instance, enactivism sees perception as a (potentially predictive) activity in its own right, generating meaning through interaction with the environment – rather than a matter of static internal representation of the external world (33).

### The 4Es approach

Enactivism has led to four important principles concerning the nature of human functioning and the mind. These are that cognition is: (1) embodied, (2) embedded, (3) enacted, (4) and emotive (21, 32, 34). This “4E” approach essentially constitutes a modern iteration on enactivism (35, 36). The landscape and the philosophical and scientific communities around enactivism and 4E approaches are complex, in particular because the “4E” approach is not synonymous with enactivism despite much crosstalk, and because proponents often incorporate of exclude different ‘e' principles when using the overarching banner [e.g., Clark and Chalmers (37), proponents of ‘extension' of mind – an alternative ‘e' principle – claim to be part of the 4E approach but not to the enactive community]. It is beyond the scope of this paper to disentangle these various approaches fully. For now, it is important to note that the theses of all 4E positions overlap significantly, as do the ‘e' principles themselves, and that as we will describe later enactivism is the most integrative of the 4Es. In psychiatry, this “4E” approach has proven valuable in understanding the mechanistic and phenomenological processes involved in psychiatric disorders. We now will briefly detail this “4E” approach in regard to psychiatric disorders (34, 38, 39).

First, the *embodied* dimension of psychiatric disorders recognizes that physical, temporal, and social embodiment in one's environment is what makes experience possible. For Gallagher (32) and Thompson (35), there is an inseparable relationship between sensation, action and environment: cognitive systems embody a dynamic sensorimotor loop. For instance, individuals move depending on their feelings, and their feelings depend on how they move (35). The physical body (e.g., sensations or sensitivity to negative events), and the subjectively experienced body are co-components. They should be considered simultaneously in the exploration of psychiatric disorders. This enactive principle could also be one of the foundations of contemporary computational theories, in particular in active inference (40).

The *embedded* dimension of psychiatric disorders means that individuals are contextually situated in their environment. An embedded approach to psychiatry means that each clinical situation should be based on the patient's context and how the client makes sense of this context (41). A patient's life experience cannot be dissociated from the environment in which her/his experience takes place. In this perspective, the expression of the paranoid delusion of a patient about his next-door neighbor can only be understood through the understanding and analysis of his home and his daily living conditions. In other words, manifestations of the disorders depend on the meaning given by the patient to her/his experience, and they can never be sufficiently described out of their cultural and social context (38, 42).

An *enactive* understanding of psychiatric disorders means that cognition is not understood to occur solely ‘inside the head' or to involve representing a pre-given world as accurately as possible. Rather, cognition – or *sense-making* – is understood as an active process, constantly unfolding as someone explores and makes sense of their environment. Under enactivism, all living systems are sense-making systems. They are autonomous, adaptive, and they regulate their own activity and exchanges with the environment, in accordance with their needs – be this a basic metabolic need for a food source or a deeply held socio-cultural value. For instance, pathological social anxiety typically represents complex feedback between attentional and behavioral engagement with social situations, heightened autonomic response, and the subsequent over-estimation of the threat of negative social evaluation, to the point that the individual struggles to source important needs from their social environment. In other words the way that someone is making sense of social situations is not helping them meet their important needs. Mental disorders are so often about something going wrong in the way we make sense of the world (42). We will therefore return extensively to this notion of sense-making.

The *emotional* dimension of psychiatric disorders under a 4E or enactive approach considers affective states as an embodied and enactive mode of evaluation by which the patient engages with and gives meaning to the world (including his/her disorder) (43). Emotions as a felt sense are seen to facilitate actions that have been adaptive or otherwise rewarded in the evolutionary or developmental past. This is congruous with but runs deeper than talk of emotions as ‘tools' for engaging with and making sense of the environment through emotional states (44, 45). For example, within evolutionary psychology, emotions are often considered as processes allowing the survival of an organism in front of a threat (46). It also roughly accords with the various different theories bearing on the definition of emotions, understood either as physiological changes for authors like William James (47), or according to the cognitive appraisals of a situation for authors like Walter Cannon (48), or as functions for processing information from the environment, for authors like Stanley Schachter (49). Enactive approaches, however, see emotions/affectivity as more than just ‘tools' that are added on top of our cognition, instead viewing cognition as thoroughly affective in nature. Giovanna Colombetti describes a *primordial affectivity*, an essential dimension of our embodied existence and not a contingent phenomenon of the mind. This affectivity would be the condition of the possibility of more specific affective states such as emotions and moods, and it is through the enactive approach that a meaning is conferred on this affectivity (50). Enactive versions of emotion are always intertwined and inseparable from experience: during an episode of paranoid-themed delirium, the person *feels* constantly threatened and emotions facilitate responses to this threat.

The enactive approach has been applied to many scientific fields in recent decades [e.g., (51–54)]. Only very recently has it been applied as a comprehensive approach to understanding clinical psychiatry (23, 42, 55). We will then detail an aspect of the enactive approach important for clinical practice: the notion of sense-making.

### Sense-making

Enaction is indeed totally applicable to clinical practice with respect to sense-making in the patient-clinician dyad (42). Psychiatric disorders are deeply entangled with values and norms (39). In this context, one of the central concepts of the enactive approach corresponds to the notion of sense-making (35, 56, 57). Sense-making corresponds to the fact that the patient, embedded in their environment, gives meaning to this environment in order to maintain their life and identity, and the alternation or loss of this sense-making is one of the keys to understanding psychiatric disorders under the enactive frame (58).

The notion of sense-making corresponds to the diversity of understandings and engagements with the world across organisms, and that how a particular organism makes sense of and engages with the world depends upon on the structure, capacities, needs, and values of the organism, as well the environment itself. For example, sense-making accounts for the fact that a substance attracts the consumer thanks to the addictive characteristics of a substance and thanks to the individual characteristics of the consumer and their history (e.g., genetics and behavioral reinforcement) (59). Similarly, postpartum blues (non-pathological) constitutes a reaction deemed normal due to a set of biological, physiological, environmental, and cultural characteristics related to a particular context, i.e., the demanding reorganization of meaning and experience in response to the appearance of new concerns related to the newborn and navigating this reorganization in light of ones culturally informed expectations regarding motherhood. In other words, experiencing some emotional turmoil or flattened mood after going through pregnancy, birth, and the sudden demand to reorganize your life around a dependent other is fairly understandable and a normal part of the process. Conversely, postpartum depression (pathological) hinders the patient's relationship to the world and to their new child: the meaning that the patient gives to the world no longer corresponds to her/his needs and values, but to a system of meaning characteristic of depression to the point that this becomes a problem for mother and baby (60). The agent is always situated within a world of meanings. However, psychiatric disorders demonstrate by contrast the loss or significant alternation of this meaning, resulting in a dysfunctional engagement with the world.

This notion of sense-making highlights also well how the enactive approach does not simply take a third person perspective where by people and the psychiatric challenges they face are simply objects of study. Rather enactivism and the notion of sense-making explicitly invites first and second person perspectives (61, 62). These intersubjective or second-person perspectives necessitate that clinical decision making should be informed not only by clinical and scientific standards, but also if reference to the cultural background, habits, beliefs and preferences of the patient. As we will discuss in the later section dealing with the limits of pluralism, such first and second person perspectives are missed within many approaches to explanatory pluralism.

### Limits of the enactive approach

One of the postulates of enactivism is that behavior is the product of complex and irreducible causal interactions across multiple scales of enquiry. This does not mean that neurobiological, behavioral or social explanations are confused or claimed not to happen. In the enactive approach, a distinction is still made, and labeling is always possible between objects and processes at different scales (e.g., genes, proteins, dendritic spine density, political parties, and cultural processes all exist). However, enactivism by itself as a perspective from philosophy of mind, does not contain the conceptual tools to analyze such processes and objects. In this way, it does not itself sufficiently account for the mechanisms and material relationships that constitute multi-scale autonomous systems to provide a pragmatic framework for psychiatry (63). Indeed, instead of explaining psychiatric phenomena in terms of mechanism, many enactive approaches seek to explain these phenomena in terms of closed networks of self-sustaining constraints (37). Such holism would seem to make it difficult to provide causal explanations of phenomena fit for psychiatry's purposes. In other words, the enactive approach to psychiatry is predominantly theoretical/conceptual in nature. To use Varela's term, enactivism constitutes a research ethics (64). As such, the ‘nuts and bolts' required for the modeling of many important aspects of psychiatric disorders are missing from enactivism itself.

In sum, enactivism sits primarily as a theory of cognition/mind (65) that does not itself provide the tools to study the mechanisms of distress/dysfunction at different levels of analysis relevant to living organisms. Such tools are necessary parts of explanatory research and clinical practice in psychiatry. Simply put, a strictly enactive or 4E approach is not enough by itself. An enactive approach to psychiatry should therefore be open to other perspectives or systems of representation. It should in other words be either itself pluralistic or be used as one way of understanding within a wider pluralistic approach.

## Explanatory pluralisms in psychiatry

### Definition of explanatory pluralism

When we speak about explanatory pluralism in psychiatry, we are referring to the simultaneous acceptance of multiple different perspectives and ways to explain mental disorders and their constituent phenomena. These perspectives may be targeted at or across any level/scale of enquiry and represent and conceptualize disorders in different ways. In psychiatry, the existence of multiple representations leads to considering different levels of understanding of life and functioning, ranging from a biological perspective to a social perspective. In the view of scientific democracy, explanatory pluralism encourages considering a set of intersecting perspectives to understand the patient. This consideration of a variety of perspectives raises the question of their integration (66–68). In other words, can we (or should we) integrate different perspectives (e.g., neurobiology, psychoanalysis, behaviorist, computational, systemic, phenomenological, sociological or anthropological approaches)? In order to answer this question, different kinds of pluralisms have been developed. Such a typology of pluralisms distinguishes non-integrative from integrative pluralisms. This will now be discussed. For clarity, we are interested here in explanatory (or “epistemological”) pluralism, which differs from an ontological pluralism that we will not discuss further.

### Non-integrative pluralisms

Non-integrative pluralism seeks to understand how different potentially incompatible or untranslatable levels of understanding, perspectives or systems of representations can coexist in a scientific or clinical landscape. It does not seek to bring together or link the different perspectives of psychiatry. For instance, it aims to question how several perspectives or levels of understanding can coexist in clinical practice, without being translated one vis-à-vis the other. At least two types of non-integrative pluralisms have been proposed: tolerant non-integrative pluralism and dappled non-integrative pluralism.

First, tolerant non-integrative pluralism has been defended by authors such as Longino (69) or Mitchell (70), with a view to promoting a division of labor between disciplines. This division would allow avoiding any form of scientific imperialism, i.e., the predominance of one perspective over the others. Tolerant pluralism considers that the choice of one perspective over another depends on the question asked and the answer expected (71–73). The choice of a neurobiological perspective can be relevant to guiding the initiation of a pharmacological treatment; the choice of a psychodynamic perspective can be relevant to understanding family dynamics in an adolescent. The relevant perspectives or level of explanation would thus depend on the epistemic and pragmatic interests of the researcher and the clinician.

The second type of non-integrative pluralism is a dappled one (74). Under dappled non-integrative pluralism, each explanation is seen to explain different aspects of the wider reality, like paint dappled on different parts of a canvas gradually revealing a wider picture. One way to explain this is in regard to the ‘laws' of scientific disciplines (for accuracies sake we should specify that given human behavior and dysfunction is rarely if ever law-like, disciplines such as psychology and psychiatry generally utilize general rules or generalized models rather than postulating laws). Laws/rules/models generally belong to certain scientific fields and apply only to these fields but looking across multiple scientific domains gives us the richest view of reality. In this way, any particular disciplines' set of laws/rules/models describes one spot of the dappled landscape of reality. Applied to psychiatry, some neurobiological rules may explain certain psychiatric phenomena, and some behaviorist rules or cognitive models help explain others. This patchwork of rules and models certainly leads to apparent disunity in the discipline, but also makes it a strength in the consideration of such a pluralism. Indeed, the scientist or the clinician can then choose the rules which best correspond to her/his objectives, in an opportunistic way. She/he can use a set of rules according to her/his will, her/his medical culture, his/her intuitions, her/his expertise, her/his relationship to risk and uncertainty, or even the institutional and social pressures exerted on him/her (75, 76).

How many different groups of rules are there? Some authors claim that this number is limited, in particular, because of the limited number of “styles” for doing science (77). Thus, only seven styles could sum up all of the past and present sciences: a mathematical style, including the geometric style and the combinatorial style, a laboratory-style (of instruments, of the creation of phenomena, of measurement), a Galilean style (of hypothetical modeling), a taxonomic style, a style of probabilities and statistical style, a “historico-genetic” style (as in geology, philology or psychoanalysis), and an experimental style (77).

In short, non-integrative pluralism recognizes that choosing one perspective on the world does not reduce the possibility of choosing others. Rather, the choice of one perspective is secondary to the consideration of all perspectives, and one is free to utilize multiple perspectives or system of representation, so long as doing so adds epistemic and/or practical value.

### Integrative pluralisms

In order to deal with the variety of representations in psychiatry, another form of pluralism has been proposed: integrative pluralism. This pluralism proposes the development of a general framework bringing together the different levels of understanding, perspectives, systems of representations, their tools and their objectives (78). Therefore, integrative pluralism aims to study how one of these levels or system can be translated into another. Unlike non-integrative pluralism, integrative pluralism does not deal with the question relative to the researcher or clinician (tolerant non-integrative pluralism) and does not consider the existence of different groups of laws (dappled non-integrative pluralism). Within integrative pluralism, for a given psychiatric disorder, there is a concentration of certain perspectives or levels of understanding (e.g., neurobiology or social influences) that can best explain the production of given clinical manifestations. Thus, the understanding of psychiatric disorders is disseminated over several levels of understanding or perspectives (74, 79).

For instance, the levels of understanding that explain the manifestations of the spectrum of schizophrenia (or even more in the case of a genetic syndrome with psychiatric expression, such as Williams syndrome) rather belong to the biological domain. Conversely, the levels of understanding that explain major depressive disorder tend to belong to the psychological (such as ruminations that maintain mood sadness) or environmental (such as detrimental social factors) domains. Finally, the manifestations of substance use disorder are better explained by all of the interacting levels: for example, in alcohol use disorder, we find levels of explanation ranging from biology (genetic variants influence ethanol metabolism), cultural factors (norms regarding alcohol consumption), psychological (certain personality traits), and social (peer availability and use) explanations (79). These levels are neither necessary nor sufficient: they influence the statistical probability of the presence of the disorder in a given individual. Environment influences gene expression and biological manifestations, and vice versa. Because of these mutual influences, such a pluralistic framework can be modeled in the form of *patterns* testifying to the conditional independence between heterogeneous variables, within symptom networks (10, 80, 81).

Among these characteristics, four factors characterize integrative pluralism: (1) the need for interdisciplinary practice in order to conceive and analyze the levels of explanation and perspectives; (2) the implication of synchronicity of the different explanations (they occur in one or more time intervals); (3) the non-exclusivity of these levels and perspectives; (4) a degree of cumulativeness (82). This last factor is particularly important because it refers to the fact that the perspectives and levels of explanation tend to accumulate over the development of psychiatry: there is no replacement of one by another, but a widening of the palette of perspectives available to clinicians and scientists (67).

### Limits of explanatory pluralism

In clinical practice and in research, it is possible to adopt a non-integrative pluralism to answer different questions, according to the needs of clinicians and researchers. An integrative pluralism, considering the entanglement of different levels of explanations, could also be interesting. However, the general framework of pluralism has limitations.

First, in its application in clinical practice, the pluralist framework is only used in a fragmented way. Such fragmentation could be partly related to the complexity and heaviness of the use of pluralism. Indeed, it involves the knowledge and manipulation of a huge corpus, almost impossible to acquire, and absolutely impossible to manage on a daily basis. In clinical practice, the use of a plurality of practices, selected according to contexts, questions and patients, requires mastering each of these practices and to know how to apply them precisely. Being loosely mastered and defined, clinicians find it difficult to apply and teach such perspectives in their entirety. Thus, clinicians can hide a certain wooliness behind their “pluralist” label, which could be just a banner made up of the perspectives it incorporates. Without the study of these perspectives, pluralism is weak. For example, often “pluralists” are not specialists in enactivism *and* phenomenology *and* biology *and* social psychiatry, etc. They are *philosophically* or *conceptually* pluralists and there are practical limitations on the breadth and depth of any clinician's knowledge and skills. Thus, clinicians cannot be ‘perfectly pluralistic' in practice, in the sense of grasping all possible scales and ways of understanding. This impossibility is sometimes managed by a simplification of the complexity at hand, which can ultimately lead to a form of managed reductionism (83). An enactive approach, meanwhile, is (or at least should be) open to multiple scales and ways of understanding yet demands that the resulting explanations be placed in the context of the embodied and meaning-experiencing organism standing in relation to its environment. Hence our interest in advocating both pluralism *and* enactivism, as we will do in the third section.

Secondly, contrary in particular to the enactive approach, first person experiences are often not directly considered in pluralistic frameworks. When conceiving of a pluralistic approach to psychiatry, it is common (but not necessary) to do so using the structure of traditional levels of enquiry (i.e., chemical, genetic, cellular, organs, and so on). However, such a conceptualization often side steps first person experience. Similarly, pluralistic frameworks often struggle to, or otherwise miss, consideration of what de Haan (42) refers to as the existential dimension of mental disorders (84, 85). For instance, when applying pluralism to major depressive disorder, there is a tendency to separate patients' sadness or anhedonia into two domains (biological or psychosocial), three domains (biological, psychological or social), or four domains (biological, psychological, social and phenomenological). However, even when phenomenological analysis is incorporated into a pluralistic approach, it is often seen as adjunct and purely descriptive, artificially separated from the other ‘domains' rather that intimately related with them (20, 42). In other words, even when it is addressed, a patient's personal experience of hearing and feeling his/her life is often seen as only one level of description in this framework, and one with little causative power. In sum, pluralistic frameworks often do not do justice to the subjective experiences of patients (4).

Similarly, the clinical application of pluralism is often deeply dualistic (86). This duality leads to a separation between the pluralist model of the clinician and the experiential model of the patient. The clinician's pluralist model can break down and localize the prejudices experienced by the patient (87), ultimately providing the patient with overly naturalistic (i.e., referring to possible cerebral dysfunctions) or overly normativist (i.e., referring to the failure of the patient's values) explanations. Value-Based Psychiatry provides a recent example of this duality (88).

## The relationship between enactivism and explanatory pluralism

### Analytical and synthetic-organizational pluralisms

Based on the discussion so far, we argue that one can simultaneously take a pluralist and enactive stance on psychiatry. This can be true in the sense that an enactive approach can be one component of a wider pluralist framework, and in the sense that (as argued by co-author KN's wider work) an enactive approach to the *conceptualization* of mental disorder can demand and incorporate a plurality of explanatory approaches (55). Enactivism can be seen as one perspective within a wider pluralistic framework, or pluralistic methods and ways of understanding can be understood beneath a wider enactive frame. Whichever way we conceive of it, nothing appears to impede this integration. Part of the originality of this article is to go further than a merely *anti*-reductionist proposal. By incorporating explanatory pluralism *and* an enactive approach, we suggest that reductionist explanations or ways of understanding can be resituated within an enactive understanding of human functioning as complex, dynamic, thoroughly affective/meaning-involving, and self-determined/operationally-closed. In this way we suggest that the utilization of both enactivism *and* pluralism, may allow for clinicians to maintain an awareness of a wider holistic/meaningful/experiential reality, without throwing away the practical knowledge that reductionist explanations sometimes have to offer.

Comparing a strict enactivism with explanatory pluralism reinforces the practical weakness of the first. A strictly enactive approach, such as descried by de Haan (42), considers that integration is necessary for a relevant and fruitful understanding of psychiatry. It is an integrative pluralism (and, as we will discuss, a synthetic one) in that it demands consideration of how the different understandings relate to the dynamic whole – a person standing in relation to their environment. Enactivism is thereby in tension with a purely non-integrative pluralism as it is constantly asking us to consider how the parts and ways of understanding them come together to dynamically constitute human functioning and experience.

It is also important to note that integrativity for different perspectives or systems of representation of psychiatry does not necessarily require integrativity for levels/scales of explanation, and vice versa. Thus, an enactive approach may integrate different levels/scales of explanation while constituting only a part of integrative pluralism. However, for psychiatry, such a non-integrative pluralism (and in particular a tolerant non-integrative pluralism) seems particularly relevant. In addition to avoiding any form of scientific imperialism (the predominance of one system of representations over the others), non-integrative pluralism allows the clinician and the researcher to be flexibly free to choose relevant perspectives according to their medical interest (e.g., diagnostic, prognostic or therapeutic), interests of the patient, or non-medical interests (e.g., administrative, social).

It strikes us that there does not seem to be a language available to describe this difference. We therefore propose that the broader framework of explanatory pluralism should be described as an *analytical* pluralism, since, at first, it tends to break targets down across levels of understanding (e.g., biological from social), before it is considered whether these different understandings can be integrated or happily *co-exist*. An enactive pluralism, meanwhile, can be described as a *synthetic-organizational* pluralism, since it demands a constant return to consideration of all levels of understanding in relation to each other, in a synthetic and organizational way. We use the term “organizational” to avoid the confusion of “synthetic” being commonly used to refer to an artificial, synthetic product. In logic, the *synthesis* allows verifying that an object (e.g., an explanation) does indeed possess the properties of the set in which it is located. In other words, enactivism is a synthetic-organizational integrative pluralism because each mode of explanation (e.g., experiential, physiological, etc.) can only have meaning by virtue of the other modes/levels and of the organism in its globality. Similar ideas can be seen in a discussion by Thompson and Varela (89) regarding the possibility for the enactive approach to try to capture “concrete wholes [body] in all their complexity,” without falling into the trap of unifying such complex phenomena under a single explanatory framework (90).

**Figure 1:**
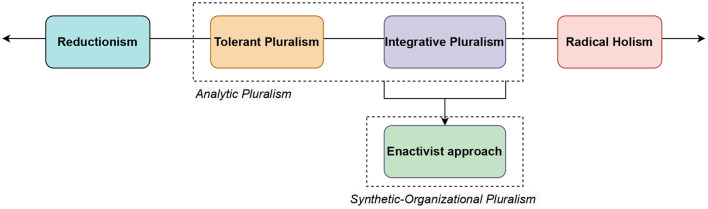
A continuum of explanatory perspectives in psychiatry from reductionism (i.e., reduction of a set of explanations to a single explanation) toward radical holism (i.e., unweighted acceptance of all explanatory perspectives). Integrative and tolerant pluralisms lie between these two poles. Enactive approaches constitute one kind of integrative pluralism, a synthetic-organizational pluralism.

To summarize, the general framework of pluralism is typically *analytical*, while an enactive pluralism can be understood as a synthetic-organizational type of integrative pluralism. Such an enactive *and* pluralistic approach to psychiatry constitutes a subtype of explanatory pluralist frameworks (here named synthetic-organizational). Figure 1 allows considering the range of explanatory perspectives of psychiatry on a continuum from reductionism to radical holism *via* integrative and tolerant pluralisms. We propose that an enactive approach constitutes one kind of integrative pluralism which can be labeled synthetic-organizational pluralism.

## Conclusion

Psychiatric practice requires understanding a variety of questions, tools, systems of representations, levels of explanation and perspectives. Reductionist approaches for clinical psychiatry can no longer be sustained. An opposing radical holism seems also untenable in practice. Psychiatry, therefore, demands to be understood pluralistically.

An enactive approach to psychiatry is beginning to emerge. It proposes that the different dimensions of understanding life and psychiatric disorders (and especially experiential, physiological, socio-cultural, and existential) are linked and integrated with each other. This stance provides an integrative conception to explain psychiatric disorders – considering their embodied, embedded, enacted and emotive (4E) dimensions. This pluralist approach is integrative and synthetic (in organizational terms) because it allows integration of different explanations and perspectives within the same theory of cognition.

A general framework of explanatory pluralism allows the simultaneous conception and the possible integration of multiple levels and perspectives within our understanding of mental disorders and psychiatry. This general epistemological approach is a broader one than enactivism and makes fewer conceptual commitments regarding mental disorder and human functioning. This potentially makes it more encompassing and flexible than enactivism as an epistemological framework, however in practice, it can often result in the glossing over of first-person experience and can allow for the importation of dualism and unhelpful eclecticism.

Subsequently, a number of perspectives should be developed, including the need to consider the second-person approach to enactive psychiatry in relation to the pluralistic framework, the issue of pragmatic choices and epistemic gains of the clinician in enactive integrative pluralism, and the intertwined understanding of enactivism as a form of pluralism or as an approach that should add pluralism.

We have here considered the relationship between enactive and explanatory pluralism. We have argued that explanatory pluralism and enactivism and mutual compatible in their application to psychiatry. We have suggested that an enactive approach to psychiatry can itself be understood as a synthetic-organizational form of an integrative pluralistic approach. In sum, an enactive approach to psychiatry has great potential as an integrative framework, but we should not give up a wider commitment to explanatory pluralism.

## Data availability statement

The original contributions presented in the study are included in the article/supplementary material, further inquiries can be directed to the corresponding author/s.

## Author contributions

CG wrote the first draft of the manuscript. CG and KN contributed to conception and design of the study. MJ and HB wrote sections of the manuscript and reviewed this work. GD wrote sections of the manuscript, supervised, reviewed, edited, and validated the work. All authors contributed to the article and approved the submitted version.

## Funding

GD received funding from the Institute for Data Valorization (IVADO) and the Fonds de recherche du Québec—Santé (FRQS).

## Conflict of interest

The authors declare that the research was conducted in the absence of any commercial or financial relationships that could be construed as a potential conflict of interest.

## Publisher's note

All claims expressed in this article are solely those of the authors and do not necessarily represent those of their affiliated organizations, or those of the publisher, the editors and the reviewers. Any product that may be evaluated in this article, or claim that may be made by its manufacturer, is not guaranteed or endorsed by the publisher.
